# Intraductal carcinoma and carcinosarcoma with bone metastases presenting in one breast: A case report

**DOI:** 10.3892/ol.2014.2573

**Published:** 2014-09-29

**Authors:** XIAN ZHONG, HONG SHEN, YING YUAN

**Affiliations:** 1 Department of Medical Oncology, The Second Affiliated Hospital, Zhejiang University School of Medicine, Hangzhou, Zhejiang 310009, P.R. China; 2 Department of Medical Oncology, Hangzhou Binjiang Hospital, Hangzhou, Zhejiang 310052, P.R. China

**Keywords:** breast carcinosarcoma, intraductal carcinoma, bone metastasis

## Abstract

Carcinosarcoma of the breast is an extremely rare and clinically aggressive tumor possessing carcinomatous components and malignant mesenchymal elements, with few cases reported in the literature. The prognosis of breast carcinosarcoma is poor when compared with more common types of breast cancer, such as ductal or lobular carcinoma. Furthermore, carcinosarcoma presents a diagnostic and therapeutic challenge due to the diversity in histology pattern and the lack of consensus regarding diagnostic criteria and effective systemic management. A 51-year-old female presented to the Department of Medical Oncology of the Second Affiliated Hospital of Zhejiang University (Hangzhou, China) with the complaint of lumbago and physical examination revealed a small mass in the right breast near the areola. Mammography and magnetic resonance imaging revealed two small masses, which were <1 cm in diameter. Positron emission tomography/computed tomography showed a maximum standardized uptake volume of 3.46 for one soft tissue nodule and multiple bones that were exhibiting radioactive enhancement. The patient was diagnosed with a breast carcinosarcoma with bone metastases and breast intraductal carcinoma. The response to chemotherapy was poor and the patient succumbed to the disease within one month of diagnosis.

## Introduction

Carcinosarcoma of the breast is a malignant sarcomatoid metaplasia of epithelial carcinoma. It is defined as a mixed tumor containing carcinomatous and malignant nonepithelial components of mesenchymal origin without a transitional zone between them. Carcinosarcoma exhibits different behavior when compared with carcinomas or sarcomas of the breast and is rare, accounting for <0.1% of all breast malignancies, worldwide (1). Bone metastasis with breast carcinosarcoma is particularly uncommon. In the current study, a case of breast carcinosarcoma and intraductal carcinoma occurring simultaneously in the right breast of a patient exhibiting multiple bone metastases is presented. Written informed consent was obtained from the patient’s family.

## Case report

**Figure 1:**
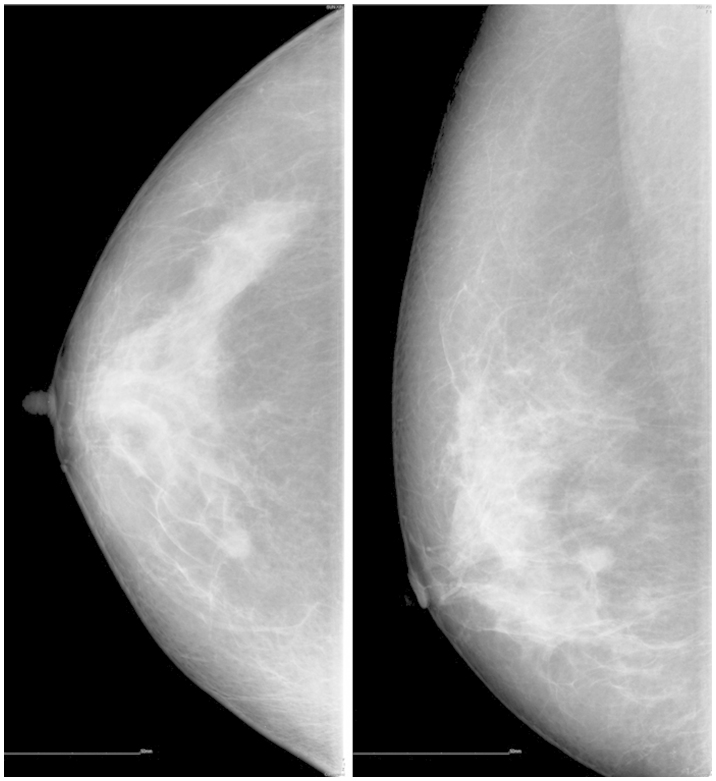
Mammography revealed two masses in the right breast.

**Figure 2:**
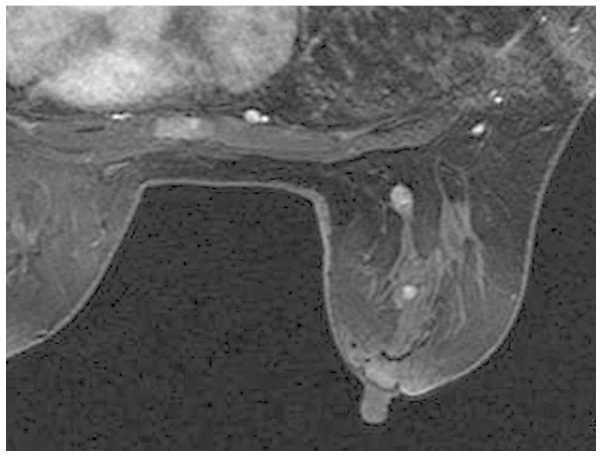
Magnetic resonance imaging revealed two nodules proximal to the areola of the right breast.

A 51-year-old Mongolian female presented to the Department of Medical Oncology of the Second Affiliated Hospital of Zhejiang University (Hangzhou, China) with the complaint of lumbago that had persisted for two months. The patient’s first-degree relatives had no history of cancer. On physical examination, a 0.5×0.5-cm mass was identified in the right breast near the areola. Bilateral axillary examination revealed no lymphadenopathy. Sonography detected two masses near the areola of the right breast; one mass measured 0.6×0.5 cm and the second mass measured 0.9×0.6 cm and was proximal to the ectopectoralis. Ultrasound examination of the bilateral axillary fossa was unremarkable. However, the patient’s mammogram was abnormal and revealed two masses in the right breast, which were situated in the upper inner and upper outer quadrants (Fig. 1). Magnetic resonance imaging (MRI) of the breast also revealed two nodules near the areola of the right breast (Fig. 2). The first node was 7 mm in diameter and located within the catheter, 30 mm from the nipple and was enhanced significantly following injection of a contrast agent. The second node was 8 mm in diameter and was proximal o the basilar section of the right breast. Positron emission tomography/computed tomography revealed an 8.1-mm soft tissue nodule with a sublobe in the upper inner quadrant of the right breast (maximum standardized uptake volume, 3.46). Radioactive enhancement was observed in multiple bones (each side of the scapula and femur, the sternum, the left eighth rib, and between the ninth thoracic and second sacral vertebrae). Based on these results, the primary diagnosis was determined as breast cancer with bone metastases.

**Figure 3:**
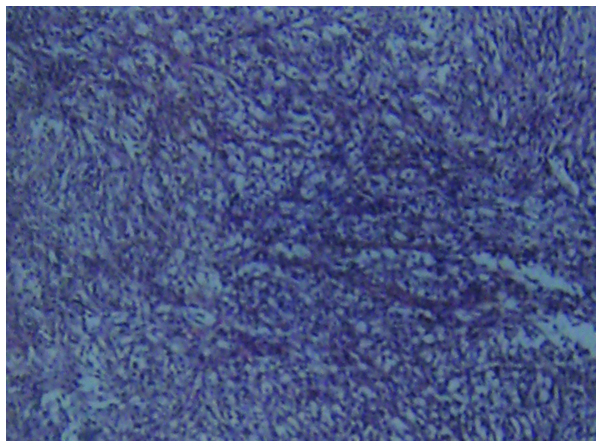
Microscopy revealed that the tumor consisted of carcinomatous and sarcomatous regions (stain, hematoxylin and eosin; magnification, ×100).

A lumpectomy biopsy was performed to verify the diagnosis. One 0.8×0.6-cm mass was identified in the upper inner quadrant of the right breast. Pathological results indicated intraductal carcinoma [estrogen receptor (ER) +, progesterone receptor (PR) +, CerbB2 −, p63 + and E-cadherin +]. The other mass (diameter, 0.5 cm) was excised from the upper outer quadrant of the right breast. Histological and immunohistochemical examinations determined a diagnosis of a malignant phyllodes tumor with invasive and poorly differentiated carcinoma (Fig. 3). Immunohistochemical examinations demonstrated no positivity for hormonal receptors or CerbB2, however, positivity was identified for vimentin [cytokeratin (CK)7 −, CK20 −, CK5/6 −, ER −, PR −, CerbB2 −, p63 +, vimentin +, cluster of differentiation 10 −, cancer antigen (CA)-125 −, CA15-3 +, CK18 +, smooth muscle actin −, gross cystic disease fluid protein 15 −, CK34BE12 −, E-cadherin +, thyroglobulin − and thyroid transcription factor-1 −]. Similar pathological results were identified by a biopsy of the fourth lumbar spinous process and the final diagnosis was determined to be breast carcinosarcoma (pT1N0M1, stage IV) according to the 7th edition of the American Joint Committee on Cancer cancer staging manual (2). One cycle of a chemotherapy regimen (lasting 21 days) comprising of cisplatin (40 mg, days 1–3), doxorubicin (70 mg, day 1) and cyclophosphamide (0.8 g, day 1) was administered. However, the patient suffered from febrile neutropenia and septic shock ten days after completing chemotherapy and succumbed after a month.

## Discussion

Breast carcinosarcoma is a rare malignancy. Patients often present with swelling of the breast or, more often with a large palpable mass on clinical examination. In rare cases, nipple discharge and retraction, or a skin ulceration may be present (3). In the patient described in the current case, lumbago was the only symptom. One small mass was palpable on physical examination. Two small masses (diameters, <1 cm) were identified by mammography and MRI. The mass had not been identified by the patient. Hematogenous spread is the most common route of metastasis, and the lungs and pleura are the most common sites of distant metastasis (4). Bone metastasis from breast carcinosarcoma has been presented in a small number of studies (5,6). Yang *et al* (6) observed bone metastasis in only one of 25 patients who had undergone surgical treatment. To the best of our knowledge, the current case is the first report of breast carcinosarcoma presenting concurrently with bone metastases. The particularly rare instance of an intraductal carcinoma presenting in the same breast was also identified.

The prognosis of breast carcinosarcoma is poor (7). It exhibits different behavior compared with carcinoma or sarcoma of the breast and is associated with a worse prognosis than classical breast carcinoma (8). The overall five-year survival rate is only 49% (9). Clinicopathological parameters, including tumor size, differentiation rate, a high histological grade, atypia and active pleomorphic spindle cells are important in prognosis (10). However, no significant difference was identified when breast carcinosarcoma was compared with high-grade receptor-negative infiltrative carcinomas (11). Hennessy *et al* (12) examined 100 patients that presented with biphasic metaplastic sarcomatoid carcinoma and 98 patients that exhibited carcinosarcoma, which were identified using the Surveillance, Epidemiology, and End Results database. The five-year overall survival rates at stages I, II, III, and IV were identified to be 0.73, 0.59, 0.44 and 0.00, respectively. The patient in the present study survived for only five months following the occurrence of lumbago.

Treatment strategies for carcinosarcoma are similar to those for breast cancer. For early stage patients, modified radical mastectomy is an efficient and practical method in the treatment of breast carcinosarcoma (13,14). Axillary dissection is usually performed during the surgical procedure, as the axillary nodes are one of the typical sites of metastasis (incidence, 26%) from either the carcinomatous or sarcomatous components (15). For patients that are late stage, chemotherapy and radiotherapy may be administered in various combinations. Evidence from existing clinical studies regarding adjuvant chemotherapy in common types of breast cancer indicates that anthracycline/taxane-based therapeutic combinations may be more effective than non-anthracycline/taxane-based chemotherapy. However, it appears that metaplastic breast cancer is less responsive to therapy that is comprised of conventional regimens, which are used for typical adenocarcinoma of the breast. Hormone therapy is not recommended due to the negative expression of hormone receptors and human epidermal growth factor receptor 2 in the majority of cases (16). In the present case, the hormone receptors stained negative during immunohistochemical analysis, and tamoxifen and aromatase inhibitors were not recommended. Due to the occurrence of multiple bone metastases, radiotherapy was not considered. The patient received palliative chemotherapy (21 day cycle) with cisplatin (40 mg, days 1–3), doxorubicin (70 mg, day 1), and cyclophosphamide (0.8 g, day 1) and the response was poor. Previous studies have indicated no survival advantage for patients treated with either chemotherapy or radiation for metastatic carcinosarcoma (17). However, the precise effects of chemotherapy or radiotherapy on breast carcinosarcoma remain unclear due to the rarity of such cases.

In conclusion, carcinosarcoma of the breast is rare and thus, few reports regarding this disease exist. Novel therapeutic agents are required to improve prognosis and further biological studies are required to identify potential molecular targets.
